# Supplementary material for: Josephson emission with frequency span 1–11 THz from small Bi2Sr2CaCu2O8+δ mesa structures
Source: Nat Commun. 2017 Nov 23;8:1742. doi: 10.1038/s41467-017-01888-4 (PMC5701082; doi:10.1038/s41467-017-01888-4)
Supplement: Supplementary file 1 — Supplementary Information [file 41467_2017_1888_MOESM1_ESM.pdf]

Supplementary note 1: Sample fabrication

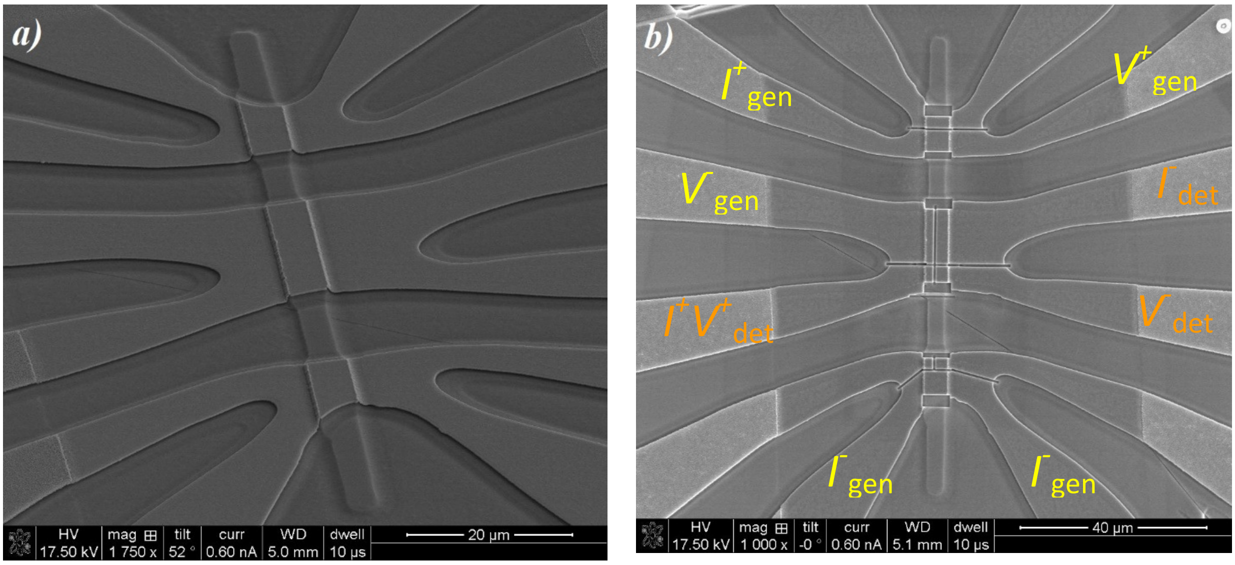

Supplementary Figure. Scanning electron microscope image of the sample before (a) and after (b) splitting into smaller mesas by Focused Ion Beam. The vertical line is the initial line-shape mesa. The crossing horizontal lines represent top metallic electrodes. In panel (a) three mesas with four electrodes each are formed at the overlap between the line mesa and the top electrodes. In (b) they are split into smaller mesas by FIB. The contact configuration used in the generator #2, detector #5 experiment is indicated in panel (b).

Sample fabrication consists of the following steps:

1. A small single crystal with an in-plane area about 200x200 μm^2 and a thickness ~10 µm is glued by epoxy to a sapphire substrate. We used Y-substituted Bi-2212 crystals $Bi_2Sr_2Ca_{0.8}Y_{0.2}Cu_2O_{8+\delta}$..
2. The crystal is cleaved and a 60 nm thick layer of gold is deposited to protect the surface of the crystal.
3. A square with dimension of 120x120μm^2 is patterned on a flat surface of the crystal by photolithography and wet chemical etching in gold etch solution (KI/I_2). The photoresist layer is removed.
4. A line shaped mesa with dimensions 5x100μm^2 is patterned on the square by photolithography and argon milling in a cryogenic Reactive Ion Etching (RIE) setup. The etching time is selected to create approximately 300 nm high line mesa in Bi-2212 crystal. The photoresist layer is not removed at this stage.
5. An insulating CaF_2 layer with a thickness of 180 nm is deposited by E-beam evaporation and a lift-off in acetone is performed to open a contact to the line mesa.
6. A planarization layer of photoresist with a window for the line mesa is made to preclude discontinuity of electrodes over sharp edges of the crystal/epoxy.
7. A sample was pre-cleaned by oxygen plasma ashing and 5 nm Ti glue layer and 150 nm gold is deposited by E-beam evaporation. After that 60 nm of Nb is deposited by magnetron sputtering.
8. A photolithography of three top electrodes with four contacts each is done on top of the line mesa, see Fig. S1 (a). Electrodes are etched with CF_4 gas (Nb) and Ar-ion-milling (Au, Ti and Bi-2212) in cryo-RIE. The photoresist is ashed away in O-plasma. After this stage three moderate size mesas (~5x10, 5x20 and 5x10 μm^2) with four electrodes each are formed at the overlap between the line mesa and top electrodes. The height of the mesas is determined by the minimum of the etching depth into Bi-2212 during fabrication of the line mesa and the top electrode etching. The top view of the sample at this stage is shown in the Supplementary Figure (a) above.
9. Finally the sample is transferred into FEI Nova 200 dual Focused Ion Beam (FIB) system and moderate-size mesas are shaped and split into several smaller mesas of different sizes and shapes, as shown in the Supplementary Figure (b) above.

Supplementary note 2: Experimental setup

Measurements are done in a cryogen-free optical cryostat with an optical access to the sample space. Each mesa could be independently biased by sending current through different electrodes. The particular contact configuration for the generator and the detector mesas, used in the discussed experiment is indicated in the Figure (b) above. The *I-V* of the generator mesa was measured in the four-probe configuration because it had two attached electrodes. The small detector mesa had only one electrode. Therefore it was measured in the three-probe configuration. The latter leads to a slope (finite resistance) at the superconducting branch in Fig. 2a. We carefully checked that there is no electrical cross-talk between the generator and the detector mesas. The crosstalk does not occur because: (i) the total current is small enough (I<200 μA) so that the base Bi-2212 crystals remains in the superconducting state; (ii) the distance between the generator and the detector is large enough to cut-off non-equilibrium quasiparticles; and also because of (iii) a specific 3D topography of mesa electrodes, V^-_{gen}, $I^+V^+_{det}$, I^-_{det}, V^-_{det},, which are placed slightly above the base crystal outside (above) the current flow path, see Fig. 1b. The absence of cross-talk is explicitly seen in Fig. 2a: application of a large bias into the generator does not lead to a shift of the detector *I-V*.

In the manuscript we present measurements with larger generators and smaller detector mesas. This is an optimal configuration because (i) The larger is the area of the generator, the larger is the total power (proportional to the area); (ii) The small detector area facilitates the highest detection sensitivity (inversely proportional to the area); (iii) A significant separation ~20 mu between the generator and the detector minimizes unwanted detector response to current injection (crosstalk), direct heating [1] or non-equilibrium effects [2-4].

Supplementary note 3: Non-equilibrium and thermal response of the detector at large bias in the generator

From Fig. 3b it is seen that at a large bias, close and above the sum-gap voltage, the detector response rapidly increases and exhibits a small maximum at $V/N=2\Delta/e \approx 30$ mV. Such a response can no longer be ascribed solely to electromagnetic waves but is also due to emission of non-equilibrium bosons that propagate from the generator to the detector through the base crystal. Analysis of such a non-Josephson emission can be found in Refs. [2-4].

At even higher bias V_{det} >7 V the detector resistance starts to decrease, which is an indication of heating that starts to decrease of the quasiparticle resistance of the detector. The zero-bias quasiparticle resistance in IJJ's has a strong, thermal-activation temperature dependence [5,6]. The increase of T leads to the reduction of R in a manner $\Delta R/R = -(U_{TA}/k_BT)\,\Delta T/T$, where U_{TA} is the thermal activation energy of the order of 30 meV. Note that the large factor U_{TA}/k_BT ~100 greatly enhances sensitivity of such a local thermometer at low T = 3 K. As mentioned in the manuscript, we do not see major reduction of the quasiparticle resistance of the detector mesa with increasing generator bias up to V_{det} ~7 V. Therefore the detector response in the bias range discussed in the manuscript is not thermal, but electromagnetic. But at a higher bias the heating starts to show up. Unlike the electromagnetic response, the heating is monotonous with respect to the dissipation power in the detector. Appearance of non-equilibrium and heating responses limits the upper frequency for our analysis to f~11 THz.

Supplementary References

[1] V.M. Krasnov, M. Sandberg, and I. Zogaj, *Phys. Rev. Lett.* **94**, 077003 (2005).
[2] V. M. Krasnov, *Phys. Rev. Lett.* **97**, 257003 (2006).
[3] V. M. Krasnov, *Phys. Rev. Lett.* **103**, 227002 (2009).
[4] V. M. Krasnov, S.O. Katterwe and A. Rydh, *Nature Commun.* **4**, 2970 (2013).
[5] S.O. Katterwe, A.Rydh, and V. M. Krasnov, *Phys. Rev. Lett.* **101**, 087003 (2008).
[6] V. M. Krasnov, *Phys. Rev. B* **79**, 214510 (2009).
